# Active Targeting of Versatile Nanocomplex Using the Novel Biomarker of Breast Cancer Stem Cells

**DOI:** 10.3390/ijms24010685

**Published:** 2022-12-30

**Authors:** Eun-Young Koh, Keun-Sik Kim, Hee-Bin Park, Jong-Seok Kim, Pyung-Hwan Kim

**Affiliations:** 1 Department of Biomedical Laboratory Science, Konyang University, Daejeon 35365, Republic of Korea; 2 Myunggok Medical Research Institute, College of Medicine, Konyang University, Daejeon 35365, Republic of Korea

**Keywords:** breast cancer stem cell, active targeting, therapeutic nanocomplex, surface biomarker, CD66c

## Abstract

Breast cancer in women is one of the most common life-threatening malignancies. Despite of the development for the improved treatment, there are still many limitations to overcome. Among them, cancer stem cells (CSCs) are well known for tumor formation, development, cellular heterogeneity, and cancer recurrence. Therefore, to completely cure breast cancer, treatment of both cancer and CSC is required. To selectively target CSCs, we generated a liposome-based smart nano complex using CEACAM 6 (CD66c) antibody (Ab), a novel cell-surface biomarker of breast-derived CSCs (BCSCs) discovered in our previous research. Selective and increased cellular uptake was observed in BCSCs treated with CD66c Ab-conjugated rhodamine-labeled liposomes (CDRHOL) depending on the expression level of CD66c. CD66c Ab-conjugated doxorubicin (DOX)-loaded liposomes (CDDOXL) selectively showed increased cell killing effects in BCSCs with high CD66c expression levels. In an in vivo animal study, CDRHOL showed enhanced accumulation in xenografted BCSC tumors with low delivery into non-target organs. Moreover, mice treated with CDDOXL have assessed the decreased induction ability of immune response by low expression levels of pro-inflammatory cytokines and reduced liver toxicity by histopathological analysis. Finally, the improved antitumor effect of CDDOXL was evaluated in a metastatic BCSC mouse model via systemic administration. Collectively, our study is the first to demonstrate that a multi-functional nano complex using a novel surface biomarker of BCSC may be a more effective therapeutic agent for the treatment of cancer and CSCs.

## 1. Introduction

Breast cancer is the second most common cause of cancer-related death in women in many OECD countries. Although its death rate has declined due to earlier diagnosis and better treatment, breast cancer is still the leading cause of death in women. More than 250 thousand individuals in the United States were diagnosed with breast cancer in 2019 [1,2]. Despite advances in medical technology and innovative treatments, such as immunomodulation and targeting strategies, they are not fully cured after treatment and still recur. Many studies have reported that cancer recurrence is caused by cancer stem cells (CSCs) [3,4].

Cancer stem cells can self-renew and promote more malignant differentiated cancer cells that offer cellular heterogeneity, playing a significant role in tumor progression, drug resistance, relapse, and metastasis in many malignant tumors [5,6]. Therefore, to completely cure breast cancer, treatment of both cancer and CSC is required. Many studies are ongoing to foster a deeper understanding of CSCs that cause the formation of breast cancer after conventional therapy. In addition, new strategies are needed to pursue new therapeutic methods and improve the diagnosis and prognosis of breast cancer patients [7,8]. However, the priority of these new strategies should be to minimize damage and unwanted action to normal cells and to treat only targeted CSCs, that is, selectivity.

There are two main strategies for the treatment of CSCs. One is the study of CSC plasticity and metabolism in terms of drug resistance and mechanisms involved in recurrence, which contributes to cancer heterogeneity [9]. The other is targeted for the delivery of selective therapeutic drugs to CSCs. Over the last few decades, many advanced approaches to address this have been developed in targeted therapy for CSCs, such as a method called passive and active targeting [10]. Passive targeting is a method of delivering drugs by the enhanced permeability and retention (EPR) effect, using the features of poorly constructed blood vascular walls around cancer cells. Active targeting, conversely, the target substance conjugated to therapeutic drugs, serves to help the drug combine well with the target cells only to show selective therapeutic effects and the increased accumulation of drugs at the target site. The mechanism of action of both methods is to eventually increase the concentration of drugs (chemotherapeutic agents, genetic materials, traditional medicine, and RNAi) into cancer cells, leading to enhanced cell death.

As delivery carriers for active targeting, nanoparticles (NPs) with target cell moieties as non-viral systems have various advantages in the field of nanomedicine [8,11]. Many types of NPs have been studied to treat cancers, including liposomes, dendrimers, polymersomes, chitosan-based nanocapsules, carbon-based NPs, inorganic NPs, cell membrane-coated NPs, and protein-based NPs [12,13,14,15,16]. Among various NPs for the delivery of drugs, liposomes, consisting of phospholipid components of cell membranes, are safe and efficient delivery systems with excellent biocompatibility [17]. Moreover, it can sustain the therapeutic effect compared to naked drugs by increasing the half-life by encapsulating both hydrophilic and hydrophobic anti-cancer drugs and therapeutics and reducing side effects to normal cells [18,19]. However, liposomes tend to be quickly removed from the blood by the reticuloendothelial system (RES). Therefore, a hydrophilic polymer (i.e., PEG) is coated on the surface of the liposome, resulting in the so-called “stealth” liposome to avoid this rapid clearance [20,21]. Therefore, it has excellent drug delivery efficiency via either passive or active targeting to solid tumor tissue.

However, no matter how good a delivery carrier is, it is useless if it does not reach the target site. Thus, again, the selectivity is highlighted here. Therefore, engineering strategies using receptors or specific surface biomarkers specifically expressed in target cells are needed to reduce side effects in normal cells and increase drug delivery efficiency [16,22]. Active targeting is achieved when this is realized. It can be employed to achieve target cell recognition and specific binding and cellular uptake, resulting in increased therapeutic potential [20,22]. For this, we have previously discovered new cell surface biomarkers, a carcinoembryonic antigen-associated cell adhesion molecule 6 (CEACAM6 or CD66c), of breast cancer-derived cancer stem cells (BCSCs) through omics-based analysis, which is the first report of its function in BCSCs [23].

The goal of this study was to demonstrate a proof-of-concept for targeted multi-functional nano complexes based on liposomes conjugated with CD66c antibody (Ab) and encapsulated with a chemotherapeutic agent for active targeting via systemic delivery against BCSCs.

## 2. Results

### 2.1. Generation of Smart Nanocomplex Conjugated with CD66c Antibody and Rhodamine Fluorescence or Chemotherapeutic Agent Based on Liposome

**Scheme 1:**
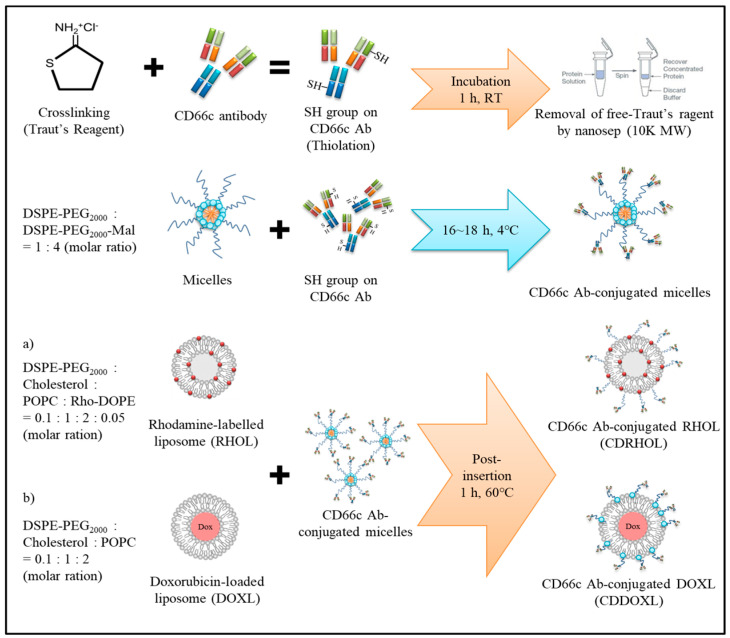
The scheme of post-insertion method for the generation of (**a**) CDRHOL and (**b**) CDDOXL.

**Figure 1:**
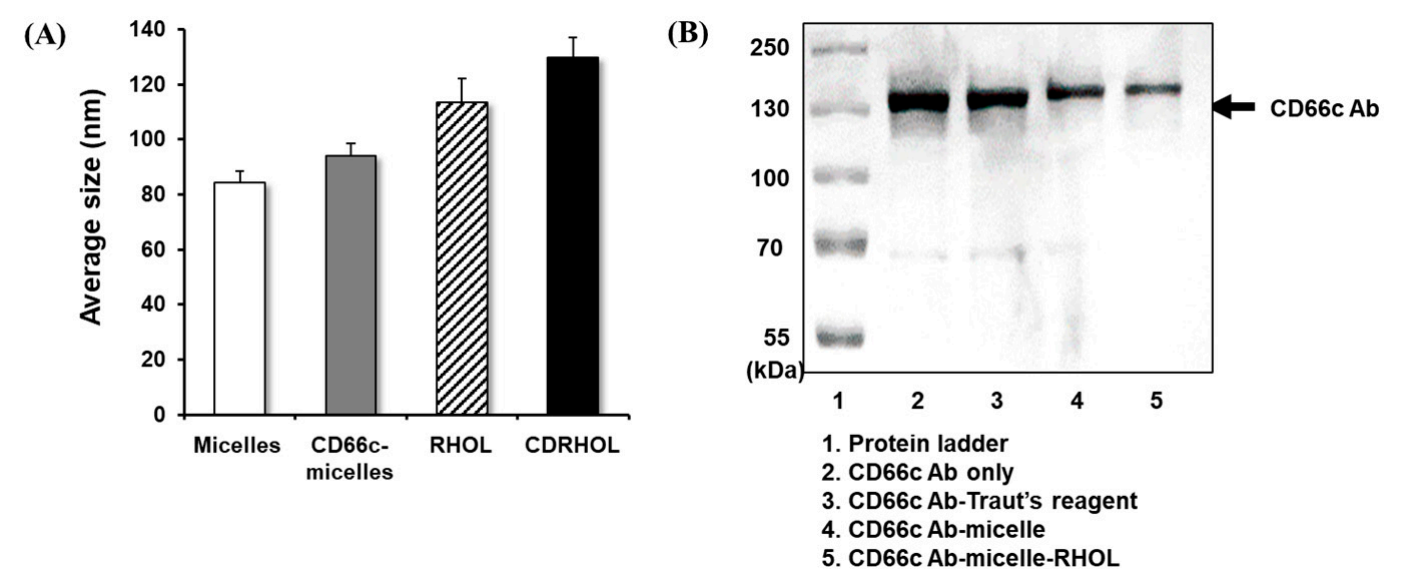
Generation and confirmation of CD66c Ab-conjugated nanocomplex. (**A**) The size of various manufactured nanocomplexes by DLS analysis. (**B**) Antibody integrity assessment by SDS-PAGE electrophoresis. RHOL: Rhodamine-labeled liposomes; CDRHOL: CD66c Ab-conjugated RHOL.

After generating chemically conjugated liposomes with CD66c Ab and rhodamine (RHO) or doxorubicin (DOX) as shown in Scheme 1, the average size of the nano complexes was assessed by dynamic light scattering (DLS) (Figure 1A). The results showed that the complex size of basal liposome with RHO (RHOL) increased from 113.3 ± 8.8 nm to 129.6 ± 7.6 nm after chemical conjugation with CD66c Ab (CDRHOL). Next, SDS-page experiments on CD66c were performed to determine whether CD66c was conjugated to the liposome surface. The IgG antibody has an average molecular weight of 150 kDa (two heavy chains of 50 kDa and two light chains of 25 kDa). As shown in Figure 1B, the molecular weight of CD66c Ab increased after conjugation because of covalent bonding with the micelle and CD66c Ab. The position of the band for the antibody in the CDRHOL (5th vertical line) was higher than that of the CD66c-micelle group (4th vertical line). These results indicate that the conjugation of the CD66c-micelle complex to RHO-liposome nanoparticles increased the diameter and molecular weight of CDRHOL.

### 2.2. Optimal Treatment Condition of CDRHOL

Compared to conventional liposomes, the induction of cellular uptake within the cell by mediating specific substances in the target cell is associated with therapeutic efficacy. Therefore, we next experimented to determine the optimal concentration and treatment time for the generated smart nano complex CDRHOL to be virtually taken up by the target cells.

**Figure 2:**
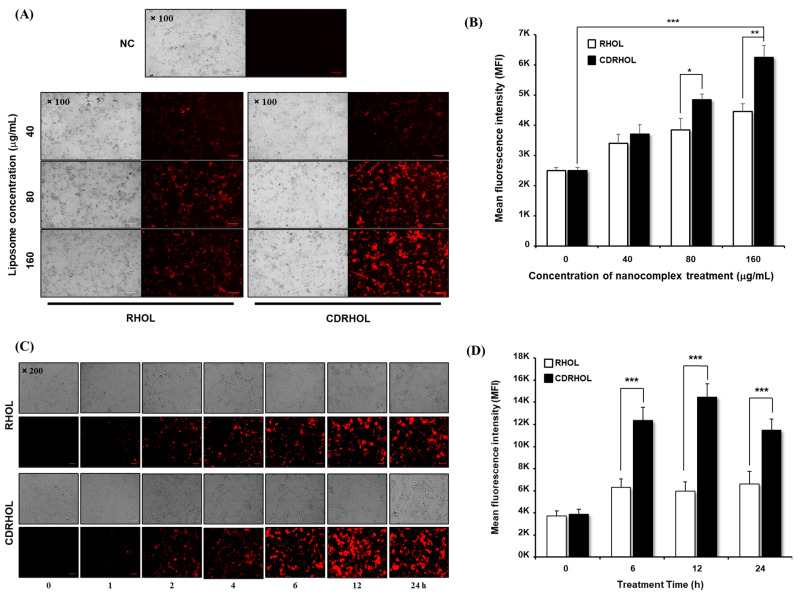
Optimal treatment concentration and time of CDRHOL. At 6 h post-incubation with nanocomplexes, the fluorescence level of the rhodamine lipids was observed and measured. (**A**) Image of cellular uptake of RHOL and CDRHOL and (**B**) Mean fluorescence intensity (MFI) of rhodamine by flow cytometer. * *p* < 0.05 or ** *p* < 0.02 for the comparison of RHOL and CDRHOL and *** *p* < 0.01 versus cell only versus 160 µg/mL of CDRHOL. After selecting the appropriate concentration, the uptake efficiency of nanocomplexes was evaluated by 1 to 24 h to select the optimal treatment time. (**C**) Uptake image of nanocomplexes according to various time and (**D**) Mean fluorescence intensity (MFI) of rhodamine by flow cytometer. Data shown represent the mean ± SD. *** *p* < 0.01 for the comparison of RHOL and CDRHOL.

The nano complexes were treated with various concentrations (from 40 to 160 μg/mL) for 6 h of incubation with BCSCs. As shown in Figure 2A, the cellular uptake efficiency in BCSCs increased from 80 μg CDRHOL treatment compared with RHOL. In particular, higher red fluorescence intensity was observed in cells treated with CDRHOL with 160 μg liposomes, indicating that the conjugation of CD66c Ab enhances the transfer efficiency of liposomes into BCSCs. The quantitative data of RHO using flow cytometry showed a more pronounced difference (Figure 2B). Based on these results, we used 160 μg liposomes as the optimal concentration of CDRHOL to target cells in subsequent experiments.

Next, we evaluated the optimal cell treatment time for liposome-based nano complexes. For this, BCSCs were treated with nano complexes, including CDRHOL, various times (from 1 to 24 h). Rhodamine fluorescence intensity proportionally increased with an increase in treatment time up to 12 h. The highest intensity was significantly observed 2.4 times higher in cells treated with CDRHOL than in cells treated with RHOL at 12 h (Figure 2C,D). Interestingly, the RHO intensity at 24 h slightly decreased. This tendency may be the result of pH condition of media, considering the results from other researchers. This is because RHO is sensitive to pH, and there have been reports that expression decreases with changes in pH [24]. Based on this result, because there was no significant difference in the uptake efficiency between 6 and 12 h groups of CDRHOL values and considering the convenience of the experiment, the treatment time for the nano complex was determined to be 6 h.

### 2.3. Selective and Enhanced Cellular Uptake of CDRHOL According to CD66c Expression

**Figure 3:**
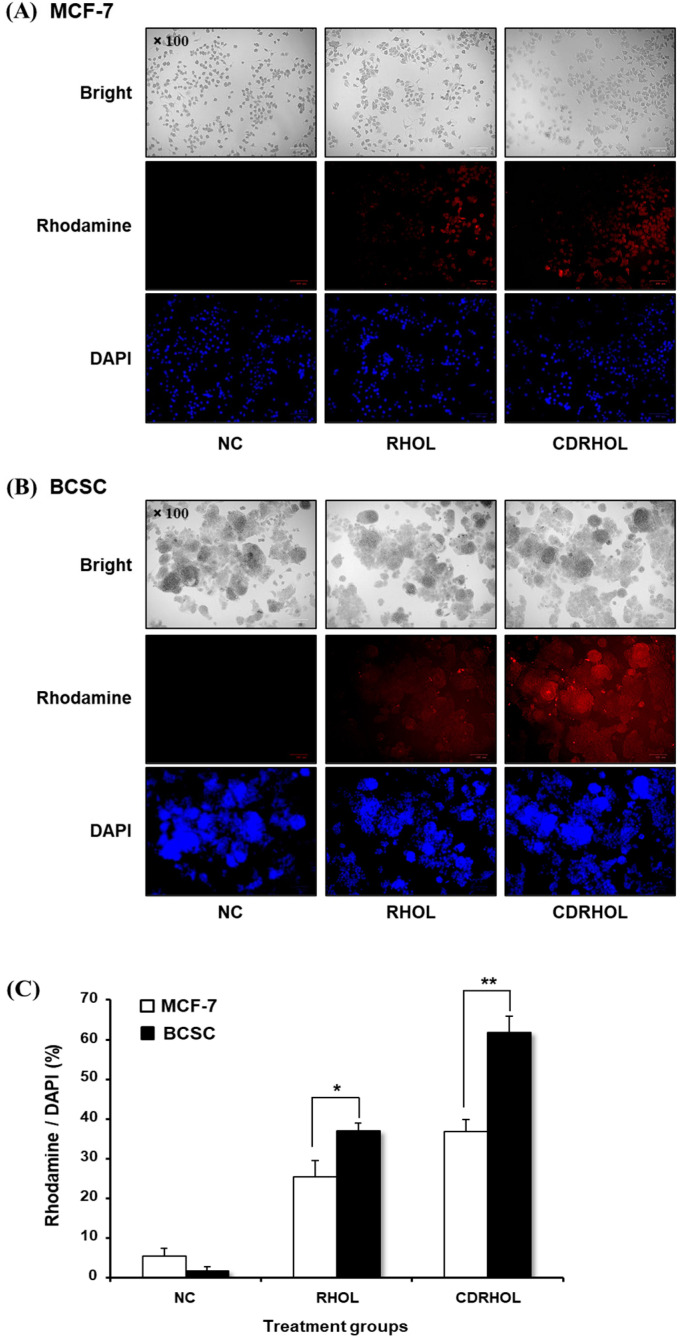
Increased cellular uptake of CDRHOL in CD66c expressing BCSC. Uptake efficiency iamges of RHOL and CDRHOL in MCF-7 cells (**A**) and BCSC (**B**). (**C**) Quantitation of rhodamine intensity of RHOL and CDRHOL uptaked into MCF-7 cells and BCSC. * *p* < 0.05 or ** *p* < 0.02 for RHOL or CDRHOL in MCF-7 versus BCSC.

Based on the previous results, we assessed whether CDRHOL increased cellular uptake with the expression of CD66c on the cell surface. CD66c high expression (BCSC) and low expression (MCF-7) cells were treated with RHOL and CDRHOL for 6 h. As shown in Figure 3A,B, RHO fluorescence intensity in RHOL-treated cells was substantially lower in both BCSC and MCF-7 cells. In contrast, cellular uptake of CDRHOL was similar to that of RHOL in CD66c low expression MCF-7 cells. However, the fluorescence intensity of CDRHOL was significantly higher than that of RHOL in CD66c high-expressing BCSCs. Rhodamine fluorescence values were quantified by normalizing the nuclei of each cell with DAPI staining (Figure 3C). As a result, it was confirmed that the cellular uptake of CDRHOL was increased in CD66c overexpressing BCSCs than in CD66c low expressing MCF-7 cells, indicating that the cellular transfer efficiency of CD66c targeting nano complex depended on CD66c cell surface expression.

### 2.4. CD66c-Specific Cancer Cell Killing Effects of CDDOXL

**Figure 4:**
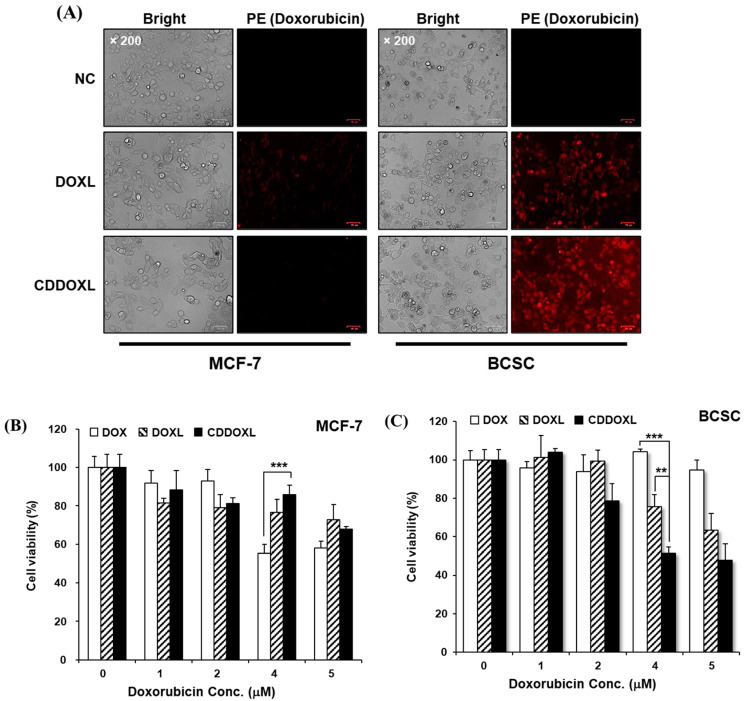
The enhanced cytotoxicity of CDDOXL in BCSC. MCF-7 and BCSC were treated with DOXL and CDDOXL for 6 h. DOX images in various nanocomplexes-treated MCF-7 and BCSC (**A**). Cancer cell killing effects of CDDOXL in MCF-7 (**B**) and BCSC (**C**). Data shown represent the mean ± SD. ** *p* < 0.02 for DOXL versus CDDOXL in BCSC and *** *p* < 0.01 for DOX and CDDOXL in MCF-7 or BCSC.

Next, we evaluated whether smart nano complexes conjugated with CD66c Ab and encapsulated with the anti-cancer drug doxorubicin (DOX) have cell killing effects according to CD66c expression. An MTT assay was performed in cells treated with DOX, DOXL, and CDDOXL at various DOX concentrations. Cellular uptake by the nano complexes was observed by DOX using fluorescence microscopy (Figure 4A). The red channel represents DOX uptake. Cellular uptake of CDDOXL was higher than that of DOXL in CD66c overexpressing BCSC. On the other hand, in MCF-7 cells, RFP fluorescence levels treated with CDDOXL were lower than those treated with DOXL. This was likely due to CD66c Ab-ablating liposomes that interact with the cell membrane and lipids of liposomes. Collectively, consistent with the previous results, this result indicates that the cellular transfer of CDDOXL into the cell works well. Therefore, we assessed whether the anti-cancer drug encapsulated in CDDOXL due to increased intracellular uptake induces a cancer cell killing effect. As shown in Figure 4B,C, both DOXL and CDDOXL induced efficient cell killing in BCSC and MCF-7 cells at high DOX concentrations compared with the NC group (0 μM). The cytotoxicity of DOXL and CDDOXL in MCF-7 cells and BCSCs increased from over 4 μM DOX. Increased cell killing was observed in BCSCs treated with CDDOXL (36.5% in 4 μM and 52% in 5 μM) compared to that in cells treated with DOXL (24.3% in 4 μM and 48.6% in 5 μM). Conversely, the cytotoxicity of CDDOXL in CD66c low expressing MCF-7 cells was not as marked as that in BCSC. Moreover, neither DOXL nor CDDOXL elicited significant cytotoxicity compared to DOX-treated MCF-7 cells. These data are consistent with the results of the cellular uptake study (Figure 3), indicating that DOX-mediated cell killing by CDDOXL specifically occurred in CD66c-expressing cells.

### 2.5. Targeting Ability of CDDOXL via Competitive Binding Assay

**Figure 5:**
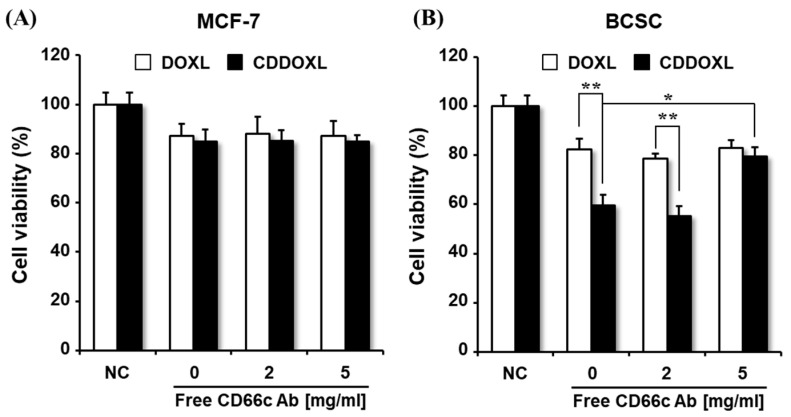
Selective specificity of CDDOXL targeting CD66c high expressing BCSC. Selective targeting efficiency of CDDOXL was carried out via competitive binding assay after CD66c Ab treatment. Cellular uptake blocking effect of CDDOXL according to CD66c Ab concentration in low CD66c expressing MCF-7 (**A**) and in high CD66c expressing BCSC (**B**). * *p* < 0.05 for CDDOXL without Ab versus with Ab, ** *p* < 0.02 for DOXL versus CDDOXL in the absence of Ab or in the presence of Ab.

In previous experiments, CDDOXL showed increased cellular uptake and cancer cell-killing effects in BCSC, where CD66c was highly expressed. A competition assay was performed to determine whether CDDOXL mediated the cell by CD66c. DOXL and CDDOXL nano complexes were treated to MCF-7 and BCSC cells post-pretreatment of two kinds of CD66c Ab concentration to address this. As shown in Figure 5, DOXL induced cell killing in both MCF-7 and BCSC without blocking the effects of various concentrations of free CD66c Ab. However, in the case of CDDOXL, cancer cell killing was decreased at a high concentration (5 μg) of Ab tested, although it was not blocked at a low concentration (2 μg). CDDOXL cytotoxicity in the presence of 5 μg free CD66c Ab was 20% lower than that in the absence of free CD66c Ab (0 μg), but free CD66c pre-treated to MCF-7 did not affect cell killing by CDDOXL. The visible blocking effect of CD66c on cancer cell killing by CDDOXL was observed in the results of Supplementary Figure S2 for blocking the cancer cell killing of CDDOXL by CD66c. These results demonstrate that the interaction mediates CDDOXL uptake between CD66c on the cell surface and CD66c Ab on the surface of the smart nanocomplex.

### 2.6. Biodistribution of CDRHOL in BCSC-Bearing Mice

**Figure 6:**
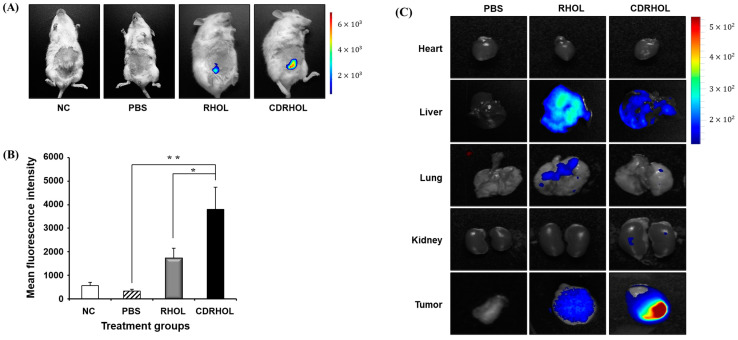
Biodistribution of CDRHOL in BCSC-bearing mice. NIG mice established with BCSC tumor were systemically injected with RHOL or CDRHOL and then at 30 min post-administration, mice were examined for various images. (**A**) Representative IVIS images of mice. (**B**) Quantification results of the signal by with an in vivo fluorescence imaging system. * *p* < 0.05 for RHOL versus CDRHOL, ** *p* < 0.02 for PBS versus CDRHOL. (**C**) Ex vivo rhodamine images of each organs extracted from mice.

Based on the in vitro results, we performed a biodistribution assay in NIG mice to assess whether intravenously injected CDRHOL accumulated into tumors. After administering PBS, RHOL, and CDRHOL to the mice bearing BCSC tumors, the accumulation of RHO was monitored using an in vivo imaging system (IVIS). Rhodamine fluorescence of the injected nano complexes was observed in the tumors (Figure 6A). An enhanced fluorescence image in the tumor region was observed in mice injected with CDRHOL. The reach efficiency to the tumor of CDRHOL was much higher than that of RHOL. The quantitative results of RHO showed similar trends (Figure 6B). After imaging, the organs (heart, liver, lung, kidney, and tumor) were extracted to determine whether nano complexes had accumulated in each organ in the body. After ex vivo fluorescence imaging (Figure 6C), the accumulation of CDRHOL was much lower than that of RHOL in most organs, especially the liver and lungs. In contrast, CDRHOL accumulated higher levels than RHOL in tumor tissue. Collectively, these results demonstrate that CDRHOL is capable of effective active targeting of CD66c expressing tumor after intravenous injection.

### 2.7. Decreased Immune Response and Hepatotoxicity in C57BL/6 Mice Injected with CDDOXL

**Figure 7:**
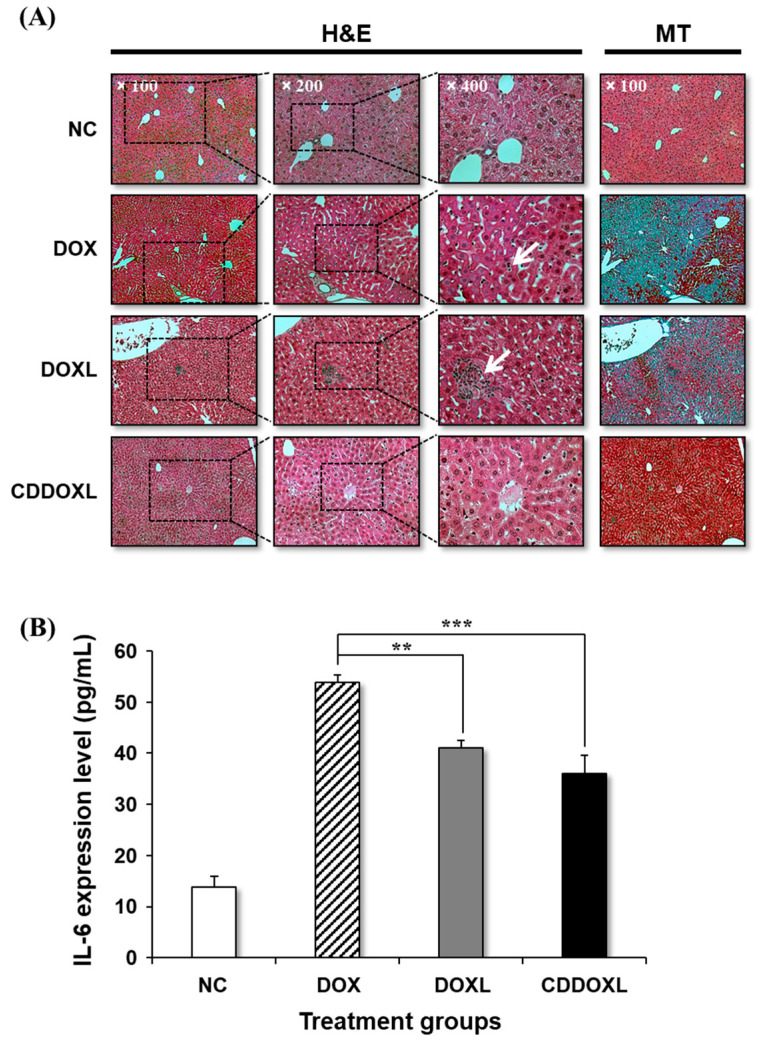
Decreased side-effects in C57BL/6 mice injected with CDDOXL. (**A**) The reduced liver toxicity and (**B**) The expression level of pro-inflammatory cytokine IL-6. ** *p* < 0.02 and *** *p* < 0.01 for the comparison of DOX-treated group and DOXL group or CDDOXL group.

One of the main disadvantages of nanoparticles being administered into the body is the induction of hepatotoxicity by liver accumulation. In a previous biodistribution profiling study (Figure 6), we observed that CDRHOL exhibited less accumulation in the liver than RHOL. These results indicated that CDRHOL induced low hepatotoxicity. C57BL/6 mice were intravenously injected with DOX, DOXL, and CDDOXL. At the same time, the induction ability of the immune response by CDDOXL was evaluated. Hepatotoxicity and the induction level of immune response were examined by H&E and MT staining of liver tissues and by the expression of pro-inflammatory cytokine IL-6 in serum at 6 h after the injection. In the histopathological analysis results of H&E staining (Figure 7A), the livers of mice injected with DOX and RHOL showed infiltrated neutrophils (white arrow), loss of sinusoids, and nuclei degeneration (Figure 7A). In contrast, no significant liver toxicity was observed in mice treated with CDDOXL compared to the NC group. MT staining revealed blue bands in the DOX and DOXL treatment groups but not in the CDDOXL group. This result indicates that the CDDOXL nano complex does not induce severe hepatotoxicity.

Next, we determined whether DOXL conjugated with CD66c has immunogenicity for innate immune system activation. As shown in Figure 7B, the administration of DOX dramatically increased serum IL-6 levels, approximately 3.9-fold higher than that in control mice. Intravenous injection of nano complexes did not significantly increase serum IL-6 levels compared to the control mice. Among them. CDDOXL decreased 1.5-fold lower than DOX-treated mice, indicating that DOX encapsulated in liposome-based nano complexes can be used safely with low immunogenicity. Collectively, these results suggest that the CD66c-targeting smart nano complex induces decreased side effects.

### 2.8. Therapeutic Effects of CDDOXL in BCSC Metastatic Tumor Model

**Figure 8:**
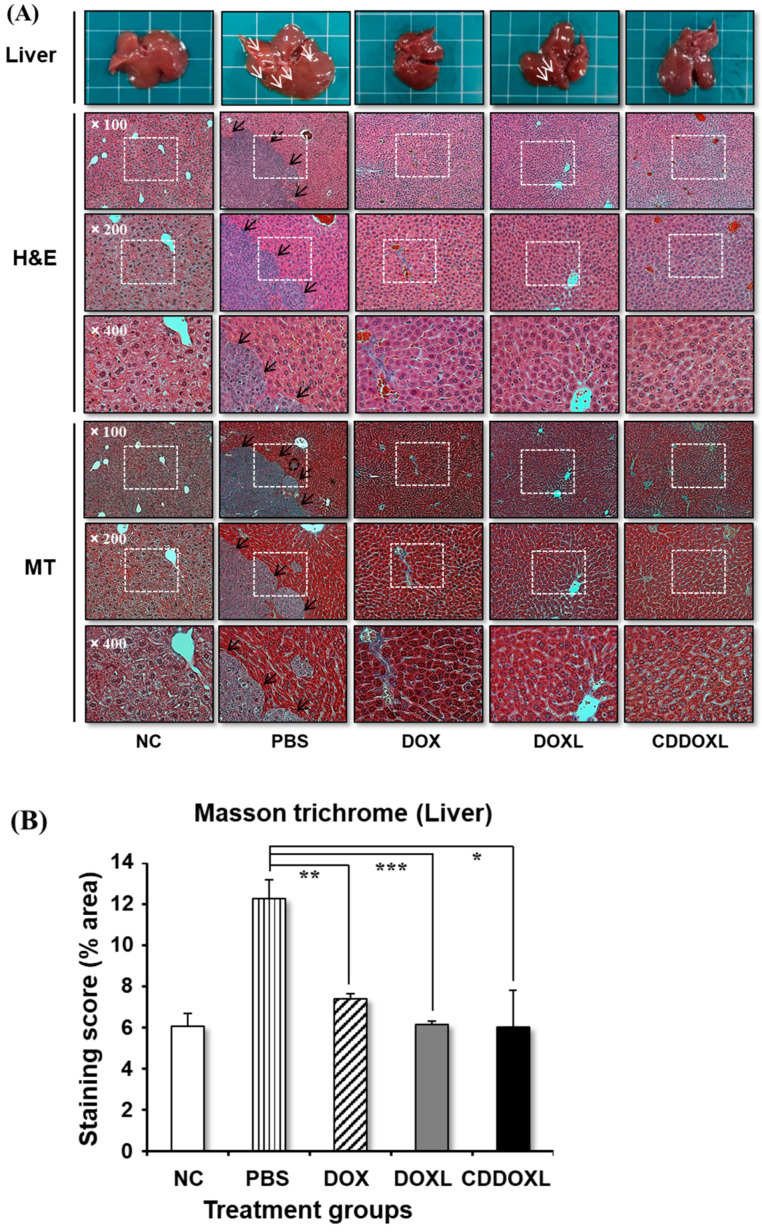

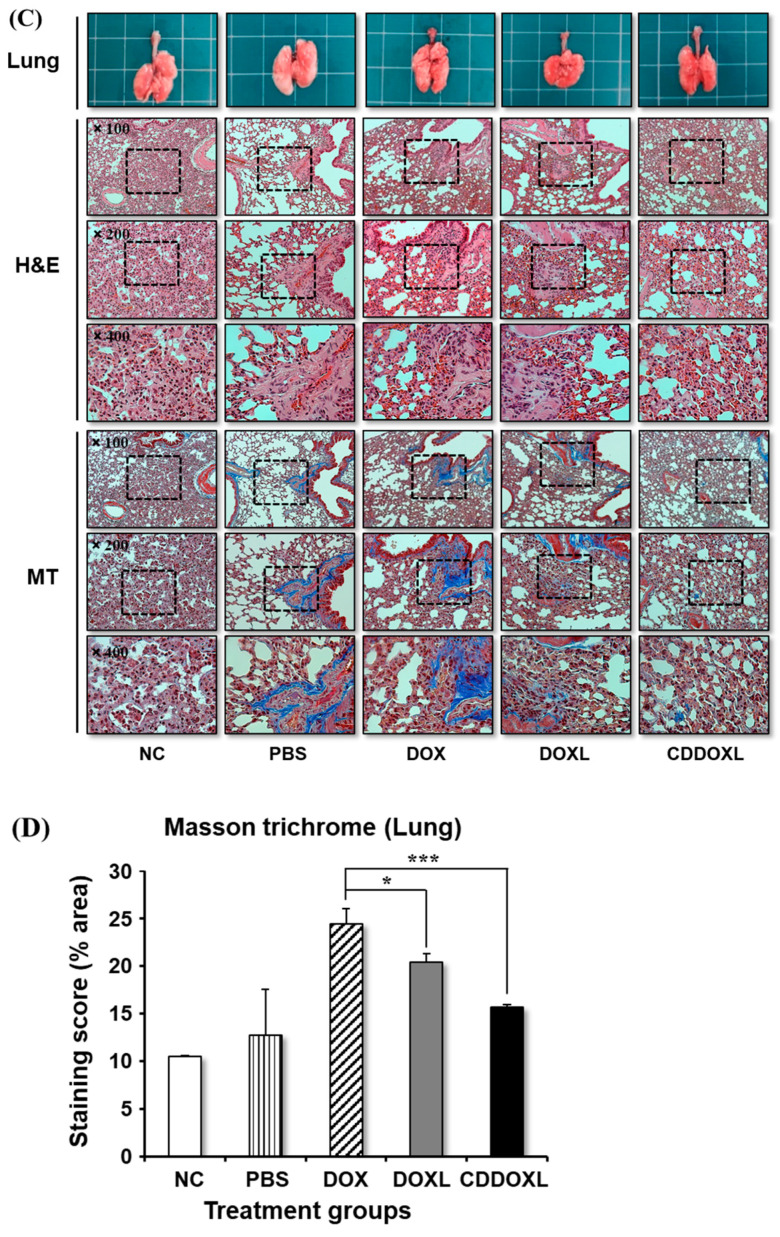
Anti-tumor effects of CDDOXL for targeted drug delivery in BCSC metastatic model. (**A**) Therapeutic effects of CDDOXL in metastatic liver cancer (white and black arrow) and (**B**) Fibrosis percentage area by masson trichrome staining in the liver of CDDOXL-injected mice. * *p* < 0.05, ** *p* < 0.02, and *** *p* < 0.01 for PBS-treated group versus DOX- or DOXL- or CDDOXL-treated group, respectively. (**C**) Therapeutic effects of CDDOXL in metastatic lung cancer. (**D**) Fibrosis percentage area by masson trichrome staining in the lung of CDDOXL-injected mice. * *p* < 0.05 for DOX-treated group versus DOXL-treated group and *** *p* < 0.01 for DOX-treated group versus CDDOXL-treated group.

CD66c-overexpressing BCSC-derived metastatic tumors were examined by observing histological modifications after intravenous injection of various Dox formulations to evaluate the antitumor activity of CDDOXL. Macroscopically, the PBS-treated group yielded many metastatic tumors as a small white spot in the liver (white arrow in the upper panel in Figure 8A), although no traces of tumors in the lungs were found. No tumor formation was observed in the free DOX- and CDDOXL-treated mice, whereas two spots were detected in the DOXL-treated mice. The histological results of H&E and MT staining, which were conducted to analyze this further, demonstrate the presence and fibrosis of cancer. According to microscopic observations of the paraffin-embedded liver tissue sections, a certain hypercellular characteristic was observed in the tumor sections by H&E staining of mice treated with PBS, compared with NC mice (middle panel in Figure 8A). Masson’s trichrome staining showed increased fibrosis due to the blue band of collagen in PBS-treated mice (bottom panel in Figure 8A). On the other hand, the other group did not exhibit any serious histological abnormalities. The quantitative values of the MT images also showed similar results to the picture (Figure 8B).

Interestingly, the results in the lung tissue showed a slightly different pattern from that of the liver tissue. When visually observed, metastatic tumors of the lung were not identified, but clear differences were observed in the histochromatological analysis. The results of the liver tissue analysis showed an exceptionally high blue band in the PBS-treated group, while the results of the lung tissue showed that the blue band was high in the DOX-treated groups. As shown in Figure 8C, H&E staining showed thickening of the inner layer around blood vessels in the lungs of mice treated with PBS, free DOX, and DOXL. In addition, overall blue bands were observed in the three groups by MT staining, leading to toxicity or metastasis to the lung. In contrast, CDDOXL-treated mice showed a similar appearance to the normal group, and the blue bands by MT staining were lower than those of the other groups. The quantitative values of the MT images also showed similar results to the picture (Figure 8D). These results demonstrate that smart nano complexes that specifically target BCSCs can reduce the toxicity of free anti-cancer drugs, inhibit the production of metastatic cancer, and enable effective drug delivery.

## 3. Discussion

The emerging trends for cancer treatments include targeted therapy and immunotherapy, including conventional therapies [25]. Under these strategies, cancer research is being studied in various ways, including the mechanism of cancer development, the discovery and substance of cancer cell signaling pathways, and the development of therapeutics using cancer cell surface materials and immune checkpoint inhibitors to boost the immune response of the host [22,26]. Despite such efforts and research, cancer remains a difficult disease to treat. The absence of fundamental treatment for cancer stem cells (CSCs), known as the cause of cancer recurrence, could be the reason why cancer is difficult to cure. Cancer stem cells remain controversial about their existence and characteristics with stem cell-like ability but treating cancer stem cells, the seeds that underlie cancer cells will be a treatment strategy for cancer [7,27].

Although anti-cancer drugs may reduce tumor size, CSCs are not completely extinct. Rather, it is known that the CSCs surviving after conventional therapy are enriched by inducing differentiation or trans-differentiation, leading to recurrence and increased sphere-forming ability for new tumor re-formation [4,28]. Therefore, the identification and targeted therapy of tumor-initiating CSCs is of significant interest in cancer research and suggests various approaches [29]. These efforts have already identified many CSC markers. For example, the following markers have been reported to target them: ALDH, CD44, CD90, and CD133 [11,30,31] for cell surface markers and Notch, Hedgehog, and transforming growth factor-β (TGF-β), which are markers of specific signaling pathways [7,32]. However, these markers tend to be highly heterozygous and lack cancer specificity, even in the same cancer. Therefore, specific targeted therapies for CSCs underline the need for a strategy based on selective targeting of CSCs.

To realize this, we identified a new biomarker, CD66c, on the surface of breast cancer-derived CSCs from previous studies [23]. We used this biomarker to enable active targeting and designed two multi-functional nano complexes based on liposomes. One is CD66c Ab-conjugated liposomes labeled by RHO (CDRHOL) to track the real-time behavior of the nano complex for the introduction of a concept called theragnosis, enabling images. The other is CD66c Ab-conjugated liposomes loaded with the anti-cancer drug DOX (CDDOXL) to enhance therapeutic efficacy. Nanosized materials in the nanomedicine field allow for drug particles or delivery devices to be manipulated at the nanoscale for improved delivery to different parts of the body while at the same time retaining the valuable pharmacological properties of the drug. Given this, the selective targeting of CSCs using biomarkers has enormous potential.

Ideally, therapeutics for cancer treatment should be able to cure original cancer and metastatic cancer. Therapeutics must be administered intravenously to achieve this. It is well known that the size of the therapeutic agent is important in the treatment of passive targeting by the EPR effect. Nanoparticles with a diameter over 400 nm do not circulate well in the bloodstream owing to their rapid elimination by the reticuloendothelial system (RES). In comparison, those with a size of approximately 200 nm are known to stay in circulation for a longer period [33]. In the present study, the generated nano complexes, RHOL and CDRHOL, did not exceed 200 nm in size (Figure 1), indicating that our particles are systemically injected and can reach the target cells through the blood vessels. In fact, the increased accumulation of smart nano complexes targeting CD66c into xenografted BCSC cancer tissues in our in vivo biodistribution study supports this fact (Figure 6). In addition, the surface of our generated nano complex was PEGylated to prevent unnecessary coupling with substances present in the blood during intravenous administration, leading to an increased opportunity to reach the target area and give the stealth function that moves away from the host immune response. Our nano complex showed low immune response induction when administered in vivo (Figure 7). Taken together, these characteristics of our nano complex mean that they will have an increased half-life, systemic circulation, and less immunogenic reaction, leading to improved anti-cancer effects.

Nanotechnology-based delivery systems have improved drug delivery efficiency at tumor sites via passive- or active-targeting mechanisms [25]. The best advantage of active targeting therapy is the selective and specific drug delivery and delivery efficiency inside target cells. This depends on biocompatible targeting moieties that bind specifically to overexpressed receptors or antigens on target cell surfaces at diseased sites and cell populations. Our smart nano complex showed high cellular uptake and drug delivery efficiency only in cells where CD66c was highly expressed (Figure 3 and Figure 4). A more clear cellular uptake efficiency according to the level of CD66c expression was confirmed through a competition assay (Figure 5). These results suggest that our smart nano complex has selectivity and potential as a therapeutic agent for targeted cells expressing CD66c.

Chemotherapy, the first generation anti-cancer drug developed so far, is used as a standard cancer treatment, but instead of having excellent anti-cancer effects, it acts on normal cells, resulting in serious side effects. The main disadvantage of using chemotherapeutic agents is the development of resistance. To solve these problems, engineered nano complexes used in nanomedicine is that therapeutic effect can be selectively focused on cancer with non-specific toxicities. As a result, a small or the same amount of drugs can produce maximum effectiveness with reduced side effects. As shown in Figure 4C, CSCs are known to be resistant to the anti-cancer drug DOX. Even when the drug concentration was increased, the cancer cell viability remained high. However, our same nano complex targeting CD66c showed better cancer cell death than DOX alone. These results indicate that targeting nano complexes encapsulating DOX can overcome drug resistance, which will have an enhanced anti-cancer effect.

In the last few decades, nanotechnology and nanomaterials have found essential roles in cancer diagnosis and treatment for better early detection and more efficient drug delivery to tumor cells [13,16] In addition to developing therapeutics with excellent anti-cancer effects, real-time tracking of therapeutics administered in vivo facilitates treatment effectiveness and prognosis determination. The in vivo traceability of nano complexes containing imaging agents or therapeutics has already been reported in many studies. From the perspective of this diagnosis, our smart nano complex with the concept of theragnosis showed selective accumulation in established tumors (Figure 6), indicating that behavioral tracking of the therapeutics is possible. In addition, from a therapeutic point of view, our nano complex was evaluated for its anti-cancer effects in a metastatic tumor model after systemic administration. Metastasis is an important factor in the prognosis of tumors [34]. The most common site of tumor cell injection employed for experimental metastasis models is the lateral tail vein in mice [35]. Tail vein injection results primarily in pulmonary metastases. In contrast, intra-splenic or portal vein injection of tumor cells is the most common site employed to develop metastasis in the liver. As the hypothesis supported by this experiment, support for the seed-and-soil effect is provided by several experimental metastasis models [36]. This hypothesis suggests that the eventual outgrowth of a tumor, in this case at a metastatic site, is defined by determinants of the tumor cell (seed) and the ability of the tumor cell to receive appropriate growth and survival signals from its microenvironment (soil). BCSC metastatic study administered intravenously to NOD/SCID mice showed successful and enhanced target antitumor effects of CDDOXL (Figure 8) via histological analysis. As a result, our smart nano complex targeting CD66c has shown the ability to reduce cancer growth by effectively eliminating cancer caused by CSCs, which are the origin of metastatic cancer. Based on these results, our CD66c targeting smart nanocomplex has a high potential for use as a targeted delivery system in cancer therapy.

## 4. Materials and Methods

### 4.1. Cell Culture

The MCF-7 human breast cancer cell line was purchased from the American Type Culture Collection (ATCC, Rockville, MD, USA) and cultured as adherent cells using Dulbecco’s modified Eagle medium (DMEM; Hyclone, Thermo Fisher Scientific, Waltham, MA, USA) supplemented with 10% fetal bovine serum (FBS; Hyclone, MA, USA) and 1% penicillin G-streptomycin solution (P/S) (Gibco, Life Technologies, Grand Island, NY, USA). The cells were maintained in a humidified atmosphere (5% CO_2_) at 37 °C. BCSCs were cultured from MCF-7 cells in 6-well ultra-low attachment plates as previously described [23]. Briefly, MCF-7 cells were cultured in serum-free Dulbecco’s modified Eagle’s Medium/F12 medium (DMEM/F12) (Gibco, NY, USA) and specific supplements including 10% fetal calf serum (Hyclone, MA, USA), 1% penicillin G-streptomycin solution (Gibco, NY, USA), 5 μg/mL insulin, 20 ng/mL EGF (Gibco, NY, USA), 20 ng/mL b-FGF (Gibco, NY, USA), and B27 (Invitrogen, CA, USA). After culturing under these conditions, MCF-7 cells were grown as nonadherent spherical cells, called mammospheres. After culturing for 14 days in a suspension state, the cells isolated by MACS were verified, and the biomarker expression (CD24-/CD44+ and CD66c expression level) was assessed by flow cytometry (Supplementary Figure S1).

### 4.2. Preparation of Micelles

**Scheme 2:**
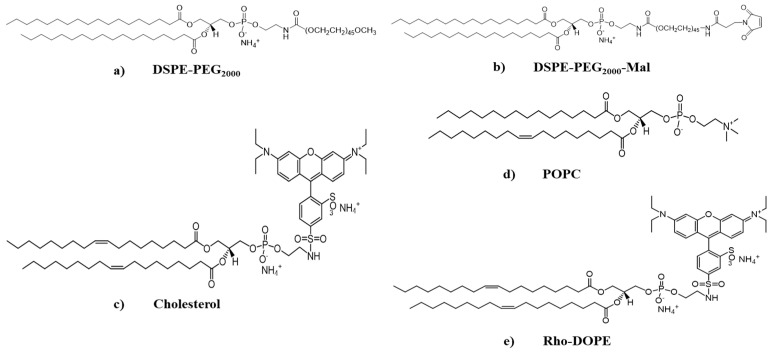
The structure of materials for the formation of micelles and liposomes based nanocomplex. (**a**) DSPE-PEG_2000_, (**b**) DSPE-PEG_2000_-Mal, (**c**) Cholesterol, (**d**) POPC, and (**e**) Rho-DOPE.

Lipid mixtures, composed of 1,2-distearoyl-sn-glycero-3-phosphoethanolamine -N-[maleimide(polyethylene glycol)-2000] (ammonium salt) (DSPE-PEG_2000_-Mal) (Avanti Polar Lipids, Alabaster, AL, USA) and DSPE-PEG_2000_ (Avanti Polar Lipids) at a 4:1 molar ratio (Scheme 1 and Scheme 2), were dried under a stream of nitrogen gas until no liquid remained. The remaining trace amounts of organic solvent were removed from the film by volatilizing the remaining solvent for 1 h in a vacuum environment. The film was then hydrated with distilled water. After hydration, the liposomes were sonicated.

### 4.3. Preparation of Rhodamine-Labeled Nanocomplex

To prepare the rhodamine-labeled liposomes (RHOL), 1,2-dioleoyl-sn-glycero-3-phosphoethanolamine-N-(lissamine rhodamine B sulfonyl) (ammonium salt) (RHO-DOPE) (Avanti Polar Lipids), DSPE-PEG_2000_, cholesterol (Sigma, St. Louis, MO, USA), and 1-palmitoyl-2-oleoyl-glycero-3-phosphocholine (POPC) (Avanti Polar Lipids) were mixed at a molar ratio of 0.05:0.1:1:2 in chloroform:methanol (2:1, *v/v*) (Scheme 1 and Scheme 2). The chloroform and methanol were evaporated under a stream of N_2_ gas and vacuum desiccated for a minimum of 1 h to ensure the removal of the residual organic solvent. The dried films (1 mg total lipids) were hydrated with 1 mL of HEPES buffer (pH 7.5) with a vortex for 5 min. The liposome solution was then extruded through 100-, 200-, 400-, and 800 nm pore size polycarbonate membranes (Whatman International, Ltd., Pittsburgh, PA, USA) to set the extrusion size.

### 4.4. Preparation of Doxorubicin-Loaded Nanocomplex

A pH gradient-driven remote loading method was employed to encapsulate doxorubicin (DOX) into liposomes (DOXL). Briefly, liposomes composed of DSPE-PEG_2000_, cholesterol, and POPC were used at a 0.1:1:2 molar ratio (Scheme 1). The dried film was hydrated in citric acid (pH 4.0) to remove the remaining trace amounts of organic solvent. One milliliter of DOXL was added to dialysis bags (2 K MWCO, Thermo Scientific, Waltham, MA, USA), immersed in HEPES buffer (pH 7.5), and incubated at 4 °C overnight. To introduce DOX into the liposomes, one milliliter of the liposome solution (1 mg/mL) was incubated with 25 μL of DOX (5 mg/mL) for 10 min at 60 °C via the difference in pH between the inside and the outside of the liposomes [37]. Finally, to remove free DOX from DOXL, the liposomes were exchanged into O_2_-free HEPES-buffer (pH 7.5) by chromatography down Sephadex CL-4B columns. To measure DOX loading efficiency in DOXL, after 45 min incubation with 10% Triton X-100 solution, fluorescence excitation and emission were measured using a plate reader (Versamax Microplate Reader, Molecular Devices, CA, USA) set at 480 and 580 nm. was used to quantify DOX in DOXL.

### 4.5. Preparation of CD66c Antibody-Conjugated Liposome by the Post-Insertion Method

**Scheme 3:**
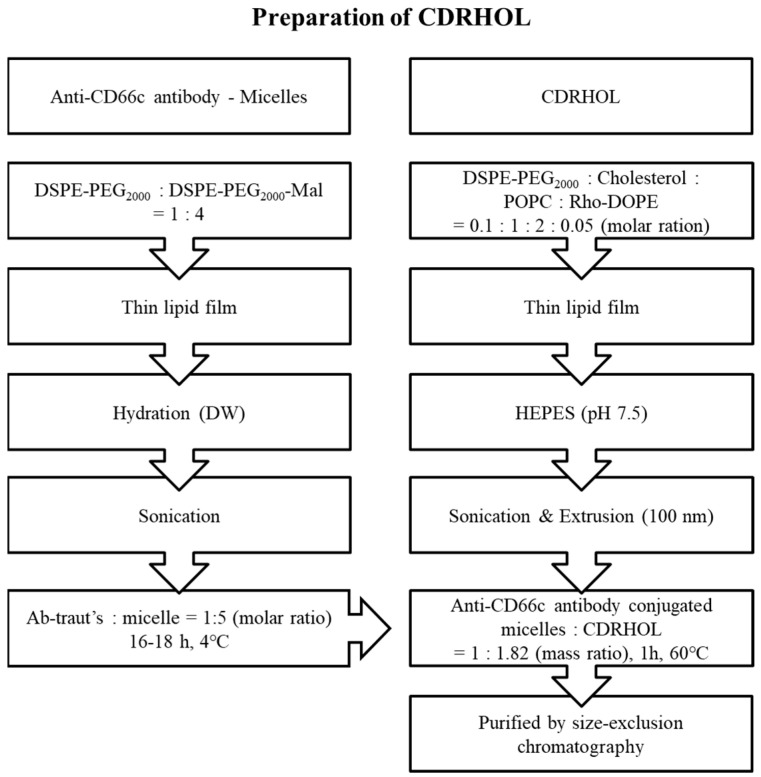
Experimental process for the production of CDRHOL.

**Scheme 4:**
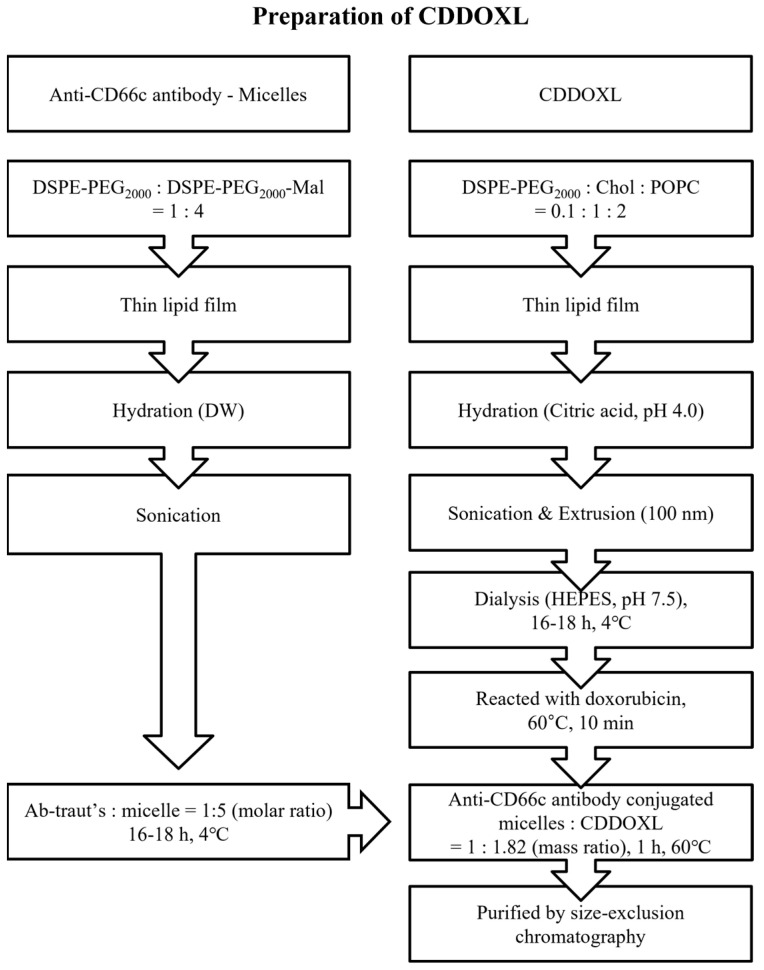
Experimental process for the production of CDDOXL.

CEACAM 6 monoclonal antibody (CD66c, 1 mg/mL, ThermoFisher Scientific, MA, USA) was thiolated by reacting with Traut’s reagent (1 mM, ThermoFisher Scientific, MA, USA). The molar ratio of Traut’s reagent: CD66c antibody was 10:1. The thiolated CD66c antibody was purified using a nanosep (10 K MWCO, VWR, West Chester, PA, USA), and the CD66c antibody recovery rate was measured using a BCA kit (ThermoFisher Scientific, MA, USA). The thiolated CD66c antibody was mixed with the prepared micelles for the post-insertion method, corresponding to a molar ratio of 1:5 (CD66c antibody:micelles) and incubated at 4 °C overnight (CD66c antibody conjugated micelles). Then, CD66c antibody-conjugated micelles were incubated with prepared RHOL or DOXL at a mass ratio of 1:1.82 for 1 h at 60 °C (for CDRHOL or CDDOXL) (Scheme 3 and Scheme 4). CDRHOL or CDDOXL were exchanged into HEPES buffer (pH 7.5) by chromatography on Sephadex CL-4B columns. Sephadex CL-4B column chromatography was also used to remove any uncoupled antibody from the liposomes. Each liposome was concentrated to 1 mg/mL lipids using a Centricon (Millipore, Bad Schwalbach, Germany). Finally, the conjugation of CD66c Ab to lipids was confirmed by gel electrophoresis. Samples were loaded on a 10% SDS-PAGE gel under reducing conditions and stained with Imperial Protein Stain (ThermoFisher Scientific, MA, USA).

### 4.6. Uptake Efficiency of Smart Nanocomplex to Breast Cancer-Derived Cancer Stem Cells

To select the optimal concentration for the treatment of generated nano complexes, BCSCs were seeded into a 48-well plate at 5 × 10^4^ cells/well and treated with 40, 80, and 160 μg/mL lipid concentration RHOL and CDRHOL for 6 h at 37 °C. To quantify the amount of rhodamine in each liposome, each sample treated with 80 and 160 μg/mL liposomes was harvested and analyzed by flow cytometry.

To determine the optimal treatment time after selecting the optimal liposome concentration, BCSCs were treated with 160 μg/mL RHOL and CDRHOL for 1, 2, 4, 6, 12, and 24 h at 37 °C. After incubation, only samples at 6, 12, and 24 h were quantified by flow cytometry, with the most visible observations of rhodamine. Additionally, to compare the cellular uptake of RHOL and CDRHOL in MCF-7 cells and BCSCs, the cells were treated according to optimal treatment concentration and time. Then, the cellular uptake was observed with a ZOE fluorescent microscope (Bio-Rad, Hercules, CA, USA).

### 4.7. Cell Viability Assay

To measure the cell-killing effects of the chemotherapeutic agent (doxorubicin;DOX) encapsulated smart nano complex, BCSCs were seeded in a 96-well plate at 2 × 10^4^ cells/well, and then the next day, the cells were incubated with free-DOX, DOXL, and CDDOXL at doxorubicin concentration of 1, 2, 4, 5 μM in a free medium for 6 h at 37 °C. After treatment, the medium was removed, and the cells were incubated for 48 h in a completely fresh medium. After incubation for 2 days, the cell viability assay was performed using MTT solution (3-(4,5-dimethylthiazol2-yl)-2,5-diphenyltetrazolium bromide) (Sigma). MTT (2 mg/mL in PBS) was added to each well and incubated for 4 h at 37 °C. The formed formazan crystals were dissolved in DMSO, and the absorbance was read at 570 nm using a microplate reader (Versamax Microplate Reader; Molecular Devices, Sunnyvale, MA, USA).

### 4.8. Competition Assay

BCSCs were seeded in 96-well plates at a density of 2 × 10^4^ cells/well. On the following day, free CD66c Ab (0, 2, and 5 μg/mL) in serum-free medium was added to the wells for 1 h at 4 °C before the addition of DOXL and CDDOXL. The cells were incubated with each nano complex for 6 h at 37 °C, the medium was removed, and the cells were incubated for an additional 48 h in a completely fresh medium. After incubation, the cell viability assay was analyzed by the MTT assay.

### 4.9. Animal Study

An animal study was performed on three kinds of mice, NOD/SCID IL2RgNULLGH (NIG) (GHBIO, Daejeon, Republic of Korea [38]), NOD/SCID (Jackson Laboratory, Bar Harbor, ME, USA), and C57BL/6 (DBL, Eumsung, Republic of Korea) to evaluate the in vivo profiling of targeted smart nano complexes. The overall design of the animal study is shown in Figure 5. The mice were retained in conformity with the National Institute of Toxicological Research of the Korea Food and Drug Administration guidelines and the regulations for the care and use of laboratory animals of the Animal Ethics Committee at Konyang University (P-20-09-A-01). All animal studies were conducted under protocols approved by the Committee on the Use and Care of Animals at Konyang University, Republic of Korea.

### 4.10. Biodistribution of CDRHOL in BCSC Tumor-Bearing NIG Mice

**Scheme 5:**
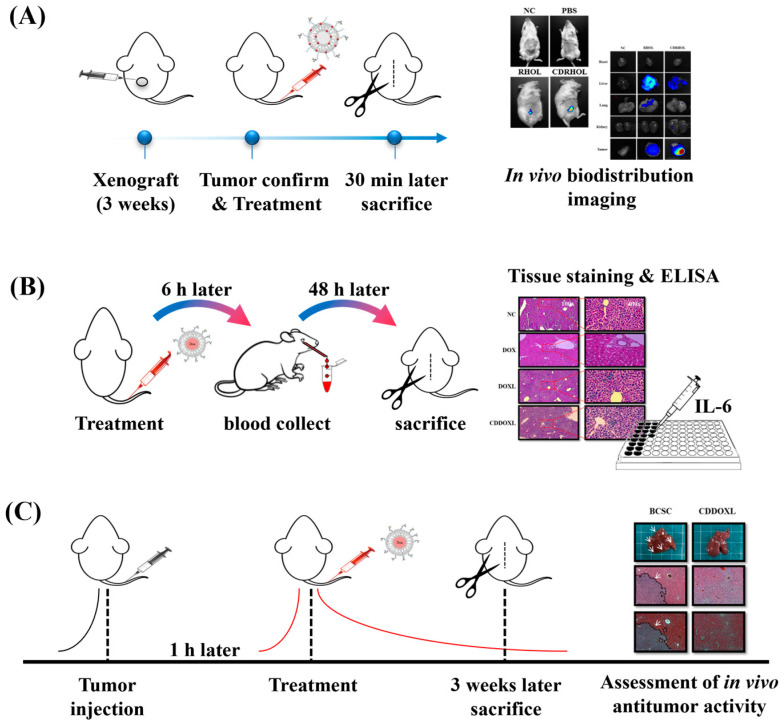
Experimental design for in vivo animal study. (**A**) In vivo biodistribution experiment of CDRHOL in BCSC-bearing mice, (**B**) Toxicity assessment study in C57BL/6 mice injected with CDDOXL, and (**C**) Therapeutic efficacy study of CDDOXL in BCSC metastasis model.

BCSCs (6 × 10^6^) suspended in 0.2 mL HBSS were injected subcutaneously into the right of the ventral side. Three weeks after BCSC injection, NIG mice bearing subcutaneous xenografted tumors were intravenously injected with the respective liposomal solutions in HBSS (RHOL and CDRHOL, 90 μg of lipid per mouse) through the tail vein. At 30 min post-injection, the mice were anesthetized with avertin (2,2,2-Tribromoethanol) and then immediately imaged using an in vivo fluorescence imaging system (VISQUE™ In vivo SMART, Vieworks, Anyang, Republic of Korea) with a PE filter composed of an excitation range of 530–570 nm and a cut-in emission filter (575–640 nm) (Scheme 5A). After imaging, the mice were sacrificed, and the organs of each mouse were extracted. The extracted organs were imaged using the same equipment to analyze the biodistribution of CHRHOL more accurately.

### 4.11. Serum IL-6 Measurement in C57BL/6 Mice

C57BL/6 mice (3 per group) were injected with either 0.2 mg/kg of doxorubicin as free-DOX, DOXL, or CDDOXL via the tail vein. Control mice were treated with PBS. Blood was collected from the mice via retro-orbital bleeding 6 h after treatment (Scheme 5B) to evaluate the hepatotoxicity of DOX-loaded liposomes. The blood was allowed to clot at 4 °C and then centrifuged for 10 min at 14,000 rpm. The concentration of IL-6 in the separated serum was assayed using an IL-6 specific ELISA kit (Mouse IL-6 Quantikine ELISA kit, R&D systems, Minneapolis, MN, USA) according to the manufacturer’s instructions.

### 4.12. In Vivo Antitumor Efficacy of CDDOXL in BCSCs Metastasis Model

The experimental metastasis model was performed as previously described, with some modifications [37]. Briefly, NOD/SCID mice were injected with a BCSC suspension containing 5 × 10^5^ cells via the tail vein. After 1 h of administration, free-DOX, DOXL, and CDDOXL containing 50 μM of doxorubicin were intravenously injected, and the livers and lungs were harvested from mice 3 weeks post-administration (Scheme 5C).

### 4.13. Histological Analysis

The extracted liver or lung organs were fixed in 10% neutral buffered formalin (NBF) before histological processing (Benchtop Tissue Processor, Leica Biosystems, Nussloch, Germany). Tissues were embedded in paraffin, and sections were stained with hematoxylin and eosin (H&E) and Masson trichrome (MT) stain. Routine histological methods were used. Images were acquired using a microscope (Axio Scope.A1, ZEISS, Oberkochen, Germany). Photographs of the organs were captured using a digital camera.

### 4.14. Statistical Analysis

The statistical significance of the data was determined by applying a paired *t*-test and one-way ANOVA. The significance of the analysis of variance is indicated in the figures. * *p* < 0.05, ** *p* < 0.02, *** *p* < 0.01. Statistical analyses were performed using the Prism version 5.01 software for window (GraphPad Software, San Diego, CA, USA).

## 5. Conclusions

Research on cancer stem cells that play a pivotal role in cancer recurrence suggests a new strategy for cancer treatment. In this study, we manufactured a liposome-based smart nano complex capable of imaging and cancer treatment using CD66c, a BCSC surface biomarker identified in our previous study. Generated smart nano complexes targeting CD66c showed selective specificity and increased cancer cell killing effect in CD66c high expressing BCSC and increased. Furthermore, these effects were observed in in vivo studies, such as the biodistribution in mice established with BCSC tumors, the induction ability of low immune response, and decreased hepatotoxicity in immunocompetent mice. In particular, the metastatic tumor model using BCSC showed significantly better anti-cancer effects than the control nano complex. Collectively, our in vitro and in vivo promising data are the first to report that our smart nano complex targeting CD66c enables selective and effective treatment for active targeting to BCSCs via systemic administration.

## Data Availability

Not applicable.

## Supplementary Materials

The following supporting information can be downloaded at: https://www.mdpi.com/article/10.3390/ijms24010685/s1.
