# Supplementary material for: Active Targeting of Versatile Nanocomplex Using the Novel Biomarker of Breast Cancer Stem Cells
Source: Int J Mol Sci. 2022 Dec 30;24(1):685. doi: 10.3390/ijms24010685 (PMC9821020; doi:10.3390/ijms24010685)
Supplement: Supplementary file 1 [file ijms-24-00685-s001.zip › ijms-2111124-supplementary.pdf]

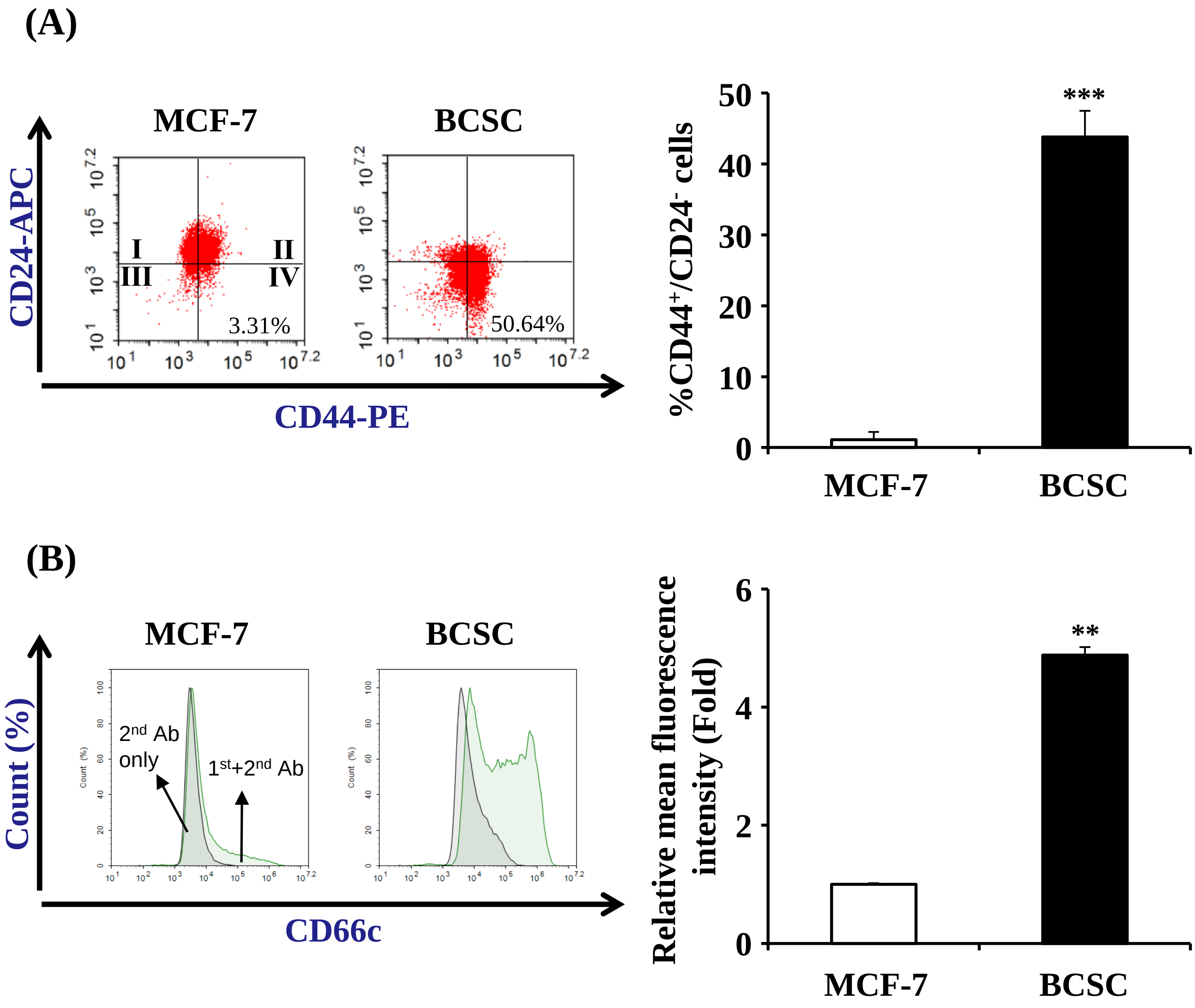

Supplementary figure S1. The characterization and verification of the isolated BCSCs. (A) The confirmation of CD44+/CD24- in MCF7 and BCSCs using flow cytometer. (B) The expression level of CD66c in each cells

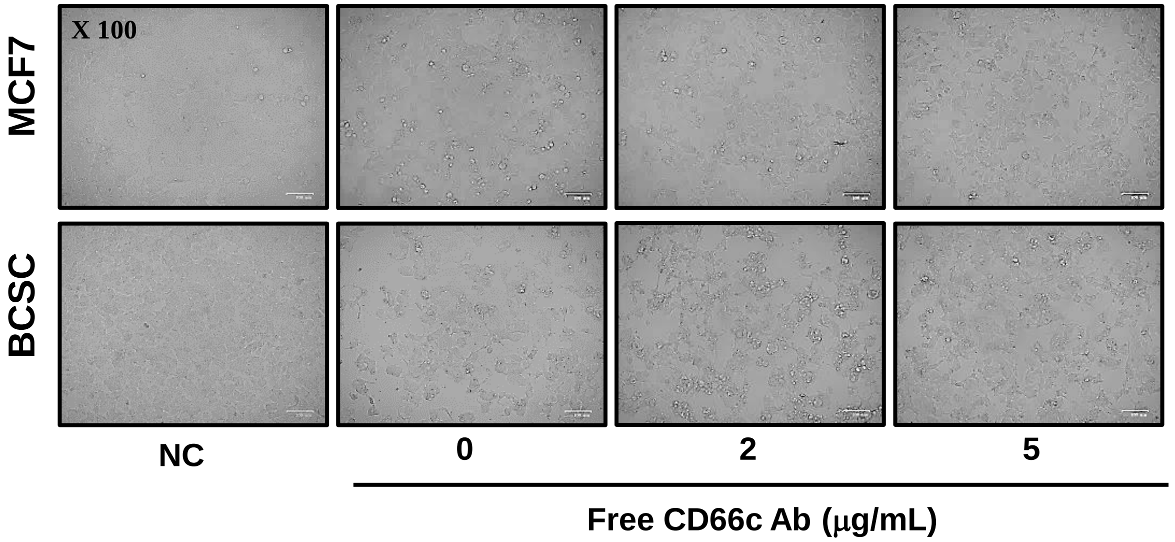

Supplementary figure S2. Competitive binding assay images of a CDDOXL according to CD66c expression.
